# A Great Meeting in Edinburgh

**Published:** 1916-05-20

**Authors:** 


### THE COLLEGE OF NURSING, LIMITED.

### A Great Meeting in Edinburgh.

### UNANIMOUS SUPPORT OF SCOTTISH MEDICAL, NURSING AND HOSPITAL REPRESENTATIVES.

A meeting of those interested in the College of Nursing was held in Edinburgh on Thursday, May 11, Dr. Barbour (President of the Royal College of Physicians) in the chair. The object of the meeting was primarily to discuss matters regarding the College of Nursing and the position of Scotland under the scheme.

The Audience.

The meeting was a very large and representative one. Among those present were Sir James Affleck, Mr. Hodsdon (President of the Royal College of Surgeons, Edinburgh), Professor James Ritchie, Dr. John Playfair, Dr. Norman Walker, Dr. McKenzie Johnston, Dr. Claude Ker, Dr. George M. Robertson, Colonel Roxburgh, Professor Glaister, Professor Ebenezer Duncan (President of the Royal Faculty of Physicians and Surgeons, Glasgow), Mr. James Macfarlane, Dr. J. Wallace Anderson, Dr. McCubbon Johnston, The Lady Susan Gilmour, Mrs. George Kerr, Miss Gill, Miss Melrose, Miss Gregory Smith, Miss J. Campbell, Miss Pegg (Dundee), Miss Edmondson (Aberdeen), Miss Thomas, Miss Wise, Miss Peterkin, Miss Rough, Miss Graham, Miss Merchant, Miss Glendinning (Falkirk), Miss Peebles (Stirling), Miss Bowhill (Perth), Miss Fraser (Elgin), Miss Shepherd (Govan), Mr. W. Barclay (Perth), Mr. W. Gray, Dr. Robert Jardine, Dr. Maxtone Thom, Mr. W. Austin (Kilmarnock), Dr. Wm. Richard (Govan), Dr. Finlay, Miss J. C. Forsyth, Miss Thyne, Dr. D. O. MacGregor, Mr. M. Morrison (Kilmarnock), Miss A. M. Milligan, Miss A. E. Rose, Miss A. M. Turnbull, and others.

The President of the Royal College of Physicians, Edinburgh.

In the course of his address Dr. Barbour pointed out that it was obvious that every question which concerned the nursing profession was of special interest to the members of the medical profession. Some would ask whether, during this time of stress and strain, the question of organisation calls for consideration, but, if it were thought out, it would be seen that it is one of the most vital questions arising out of the war. One of the great gifts which our country had given to this world had been the modern nurse—(hear, hear)—and she was born, so to speak, in the Crimean War, with which the name of Florence Nightingale has always been associated. To-day we were in the heart of a much more extensive and still more devastating war, in which the services rendered by the nursing profession would be one of the most brilliant events to look

For Matrons' and Sisters' Appointments see page 180.

back upon when the history of the war is written and we are beginning to look forward to the future.

Professor Glaister.

Professor Glaister next spoke about the College of Nursing and the objects of the Conference. He outlined the efforts made in former years to obtain State registration for nurses, and the history of the Bill which was before Parliament in 1914, and referred to the opposition which was brought forward against that Bill. He pointed out the similarity between the aims of the College of Nursing and of the Bill for State Registration, and stated that the cardinal points in both were the same.

Scottish Local Management Essential.

A discussion followed, chiefly on the Scottish Board and the local management of affairs, in which several of those present took part. The general expression of opinion was that London was too far away, that England does not understand Scottish conditions, that therefore a Local Board to manage Scottish affairs is necessary, and that a scheme which would be entirely governed from England would be unacceptable to Scotland. As one speaker said, "It seems to me that State registration of nurses is a most excellent thing, but I am quite sure that unless Scottish local management were introduced the scheme would not be a success."

Mental Nurses in Scotland.

The position of mental nurses was brought forward by Dr. Robertson, of the Royal Asylum, Edinburgh. He stated that the organisation of mental nurses was very nearly perfect. There was a register of all the mental nurses in the country, one certificate was granted, and the nurses had a three years' training. He stated that the Association permitted certificated hospital nurses to take their mental certificates after two years' training, and spoke of possible reciprocity. One of the audience raised the question of an entirely separate Scottish organisation, but this found no support.

United Action Agreed to.

Professor Glaister and Miss Gill then answered several questions, and the discussion was brought to a close by Sir James Affleck proposing that a committee be nominated to consider the appointment of a Scottish Board and to draft a constitution. This was carried unanimously, and it was decided that the committee should report to the next general meeting, the date of which was fixed by the Chairman. The meeting then closed with a vote of thanks to the Chairman, moved by Colonel Roxburgh.

